# Reply to the Comment on: Marco Cirillo The Memory of the Heart. *J. Cardiovasc. Dev. Dis.* 2018, *5*, 55

**DOI:** 10.3390/jcdd6010001

**Published:** 2018-12-20

**Authors:** Marco Cirillo

**Affiliations:** Heart Failure Surgery Unit, Cardiac Surgery Unit, Cardiovascular Department, Poliambulanza Foundation Hospital, Via Leonida Bissolati 57, 25125 Brescia, Italy; marco.cirillo@poliambulanza.it; Tel.: +39-030-351-8088

I was disappointed by Anderson’s comment on my recent review, a comment that I think derives from a too quick reading of the article. So quick that it ends up being incorrect.

When I speak of “the memory of the heart” I do not intend to refer to the Torrent–Guasp theory and I much less based my “entire review” on this theory. Carefully reading Chapter 8, the list of parameters that “the heart remembers” includes 6 (six) subjects: laminar flow, sequential electrical activation, elliptical shape, torsion, and the presence of valves with a special mention for the mitral valve.”

The graphical abstract also clearly depicts and lists all these parameters.

As a scientist, I appreciate the various theories expressed regarding the three-dimensional structure of the myocardium, because each one carries a piece of knowledge. We should not forget that the theory of the myocardial band is the only one to give a particular reading to the diastolic phase of the cardiac cycle. There is not yet a theory capable of explaining the real structure of the myocardium, as Anderson himself states [1] in the abstract of his recent review (“The precise nature of packing together of the cardiomyocytes within the ventricular walls has still to be determined”). Probably, the truth is an integration of different theories.

As a cardiac surgeon, I do not subscribe to any particular theory, I only recognize the presence in the thickness of the myocardium of bundles of differently-oriented fibers with a helical pattern. This is confirmed by the demonstration of the restoration of ventricular torsion after my reconstructive surgery technique, which redirects the orientation of the fibers in the left ventricle. I believe this is a very important piece of evidence to be translated into basic science (in addition to bringing the mortality of these patients to zero in my series of this end-stage disease).

From an expert in this field such as Anderson, I would sincerely have expected greater recognition of this particular result, which has been obtained for the first time in the history of cardiac surgery, namely to restore torsion after many years of dysfunction by putting together the residual tissue, which remains structurally intact, using a surgical suture. Patients go from 25% to 45% ejection fraction, the end-systolic volume is halved, the brain natriuretic peptide goes from 4000 to 24, and the apex returns to rotating counterclockwise.

This is a truly unique and unexpected evidence.

Every day I operate on hearts that have lost laminar flow, the geometry of the chamber, and the normal arrangement of the fibers, have stenotic or regurgitant valves, and enormous volumes compared to physiological ones. We cannot always restore these parameters and this limits the outcomes, because the heart works acceptably and efficiently with all these parameters corrected. Therefore I wonder if we can integrate the list of parameters that measure good heart performance (we have always relied only on the ejection fraction) and use them to evaluate the outcome and tune the guidelines.

The implied concept refers not only to what the heart remembers, but to what we must not forget about its physiology.

With regard to the figure from Pettigrew, the similarity (please note the term: similarity) between it and Torrent–Guasp’s representation seemed interesting enough to me, not to corroborate his theory, but to demonstrate the concept of “scientific memory” that I cited at the beginning of the review and that Anderson probably missed. The existence of almost the same image, one hundred years before Torrent–Guasp’s work and before formalin was released—this seemed like a distinctive piece of scientific knowledge to report in my review, according to the educational purpose set out by the editorial rules.

In this Special Issue of the *Journal of Cardiovascular Development and Disease*, “Functional Morphology of the Developing and Mature Cardiovascular System”, another review was published before mine [2], by one of the major supporters of the myocardial band theory. Did Anderson directly criticize him for supporting this theory with an equally negative comment?

I am very sorry that Anderson missed the meaning of my review and reduced it just to something that went against his personal opinion on the subject. Moreover, the tone of his review, which could be described as resentful, remains difficult to understand.
